# Medications as a Potential Source of Exposure to Phthalates in the U.S. Population

**DOI:** 10.1289/ehp.11766

**Published:** 2008-10-07

**Authors:** Sonia Hernández-Díaz, Allen A. Mitchell, Katherine E. Kelley, Antonia M. Calafat, Russ Hauser

**Affiliations:** 1 Department of Epidemiology, Harvard School of Public Health, Boston, Massachusetts, USA; 2 Slone Epidemiology Center at Boston University, Boston, Massachusetts, USA; 3 National Center for Environmental Health, Centers for Disease Control and Prevention, Atlanta, Georgia, USA; 4 Department of Environmental Health, Occupational and Environmental Medicine and Epidemiology Program, Harvard School of Public Health, Boston, Massachusetts, USA

**Keywords:** coating, didanosine, medications, mesalamine, omeprazole, phthalates, theophylline

## Abstract

**Background:**

Widespread human exposure to phthalates, some of which are developmental and reproductive toxicants in experimental animals, raises concerns about potential human health risks. Underappreciated sources of exposure include phthalates in the polymers coating some oral medications.

**Objective:**

The objective of this study was to evaluate whether users of phthalate-containing medications have higher urinary concentrations of phthalate metabolites than do nonusers.

**Methods:**

We used publically available files from the National Health and Nutrition Examination Survey for the years 1999–2004. For certain survey periods, participants were asked to recall use of prescription medication during the past 30 days, and for a subsample of individuals, the urinary concentrations of phthalate metabolites were measured. We *a priori* identified medications potentially containing phthalates as inactive ingredients and then compared the mean urinary concentration of phthalate metabolites between users and nonusers of those medications.

**Results:**

Of the 7,999 persons with information on urinary phthalate concentrations, 6 reported using mesalamine formulations, some of which may include dibutyl phthalate (DBP); the mean urinary concentration of monobutyl phthalate, the main DBP metabolite, among these mesalamine users was 50 times higher than the mean for nonusers (2,257 μg/L vs. 46 μg/L; *p* < 0.0001). Users of didanosine, omeprazole, and theophylline products, some of which may contain diethyl phthalate (DEP), had mean urinary concentrations of monoethyl phthalate, the main DEP metabolite, significantly higher than the mean for nonusers.

**Conclusion:**

Select medications might be a source of high exposure to some phthalates, one of which, DBP, shows adverse developmental and reproductive effects in laboratory animals. These results raise concern about potential human health risks, specifically among vulnerable segments of the general population and particularly pregnant women and children.

Phthalates, diesters of 1,2-benzenedicarboxylic acid (phthalic acid), are a group of synthetic chemicals with a wide spectrum of industrial and commercial applications. Phthalates can be used as plasticizers in polyvinyl chloride plastics, to hold scent and as solvents in personal care products, and in food packaging and processing materials (Agency for Toxic Substances and Disease Registry 2001; Hauser and Calafat 2004). Data from the National Health and Nutrition Examination Survey (NHANES) show that most of the U.S. population is exposed to phthalates [Centers for Disease Control and Prevention (CDC) 2005, 2008; Silva et al. 2004a].

Some phthalates readily cross the placenta and are developmental and reproductive toxicants in laboratory animals (Saillenfait et al. 1998). For instance, prenatal exposure of rats to dibutyl phthalate (DBP) and di(2-ethyl-hexyl) phthalate decreased fetal testis testosterone and insulin-like factor 3 biosynthesis by Leydig cells, and increased the incidence of cryptorchidism and hypospadias among male offspring (Borch et al. 2006; Gray et al. 2000; Parks et al. 2000). Despite evidence of widespread human exposure and developmental toxicity, epidemiologic studies of male urogenital tract developmental anomalies in relation to prenatal exposure to phthalates are limited. In one of the few reports, Swan et al. (2005) found a decrease in anogenital distance, a marker of feminization of the perineum, among male infants with prenatal exposure to background levels of phthalates.

Low-molecular-weight phthalates [e.g., diethyl phthalate (DEP) and DBP] are used to make coatings for oral medications, including those designed for timed release or release in the large bowel (Hauser and Calafat 2004; Koch et al. 2005). In a recent case report (Hauser et al. 2004), we identified one man with a urinary concentration of monobutyl phthalate (MBP), the main DBP metabolite, that was two orders of magnitude higher than the 95th percentile reported in the U.S. population (1999–2000 NHANES). The DBP intake of 224.3 μg/kg/day, estimated from the urinary concentration of MBP (David 2000; Koch et al. 2003), was higher than the U.S. Environmental Protection Agency (EPA) reference dose (RfD) for DBP (100 μg/kg/day) (U.S. EPA 2008). The likely source of DBP exposure was Asacol (mesalamine with enteric coating of DBP) used to treat the patient’s ulcerative colitis (Hauser et al. 2004). The association between use of phthalate-containing medications and urinary concentrations of phthalate metabolites has not been explored for other pharmaceuticals or other specific phthalates. We therefore evaluated whether such associations may be present by using data from NHANES.

## Materials and Methods

NHANES is a periodic health examination survey conducted by the National Center for Health Statistics (NCHS) of the CDC (McDowell et al. 1981; NCHS 1994). Respondents signed an interview consent form agreeing to participate, and the NCHS obtained institutional review board approval to conduct the survey. NHANES is supported by the U.S. government, and public-use data files and their documentation are available from the the NHANES website (NHANES 2008).

For certain survey periods, participants were asked if, in the past month, they had taken a medication for which they needed a prescription. The interviewer entered the medication name and selected the best match from a computerized list of prescription drugs. All reported medications were converted to their standard generic ingredient name for public data release (i.e., no specific brand names or formulations are available). The interviewer also recorded the presence of the container during the home interview and asked, “For how long have you been taking it?” Although questions about nonprescription pain relief medicines were asked in the analgesics subsection during certain periods, neither the specific brand names for analgesics nor information on other non prescription medications were recorded.

For each survey cycle, urine samples were collected from a one-third sub sample of individuals ≥ 6 years of age. Since 1999, the urinary concentrations of several phthalate metabolites have been measured at the National Center for Environmental Health using solid-phase extraction coupled to high-performance liquid chromatography–isotope dilution tandem mass spectrometry, as described previously (Blount et al. 2000; Kato et al. 2005; Silva et al. 2004a, 2004b). The phthalate metabolites for which urinary concentration data are available since 1999 are monoethyl phthalate (MEP), MBP, mono cyclohexyl phthalate (MCHP), mono(2-ethylhexyl) phthalate, mono-*n*-octyl phthalate (MOP), monobenzyl phthalate, and monoisononyl phthalate (MNP). Urinary concentrations of additional metabolites became available during later survey years. For example, the urinary concentrations of mono(3-carboxypropyl) phthalate (MCPP), monoisobutyl phthalate, mono(2-ethyl-5-hydroxyhexyl) phthalate, mono(2-ethyl-5-oxyhexyl), and monomethyl phthalate have been measured since 2001, and of mono-(2-ethyl-5-carboxypentyl) phthalate since 2003. We do not present MCHP, MOP, and MNP data because these metabolites were infrequently detected (< 20% of the samples). For concentrations below the limit of detection (LOD), we imputed a value equal to the 
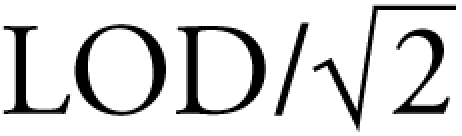
 (Hornung and Reed 1990).

We estimated the daily exposure to DBP from the urinary concentrations of MBP using the method proposed by David (2000), as expressed by Koch et al. (2003):


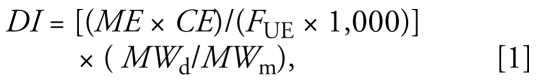


where *DI* is the daily intake in milligrams per kilogram per day; *ME* is the creatinine-corrected urinary metabolite concentration in micrograms per gram; *CE* is the creatinine clearance rate, normalized for body weight, in milligrams per kilogram per day; *F*_UE_ is the molar conversion factor that relates urinary excretion of metabolite to diester ingested; and *MW*_d_ and *MW*_m_ are the molecular weights of diester and metabolite, respectively. For these calculations, we set CE at 20 mg/kg/day for adults and used the 0.69 reported for MEP/ DEP as the *F*_UE_ value (David 2000; Koch et al. 2003).

For the present analyses, we used three files from NHANES 1999–2004 (NHANES 2008): *a* ) sample demographics file, which provides selected demographic variables such as sex and age; *b*) the prescription medication section of the Sample Person Questionnaire; and *c*) the urinary phthalates laboratory files, which include data on urinary concentrations of specific phthalate metabolites. These files were linked using the unique survey participant identifier number (NHANES 2005). Because only survey participants ≥ 6 years of age were eligible for the laboratory subsections, we restricted the analyses presented below to persons ≥ 6 years of age.

We had *a priori* identified medications that may contain phthalates as inactive ingredients using publically available sources. We first created a list of medications of interest based on their likelihood of containing phthalates [e.g., the *2006 Red Book* list of “Drugs That Should Not Be Crushed” (Thomson PDR 2006)] and then obtained detailed information on their formulations, following a thorough strategy that ranged from searching Food and Drug Administration websites and product labels to direct requests to manufacturers of both medications and phthalates. Given the limited data available in NHANES, we had to restrict the scope of the analysis to phthalate-containing prescription medications for which we were able to identify users in the study population. Further, because only some brands with a given active ingredient might contain phthalates, we selected medications for which phthalate-containing brand(s) were likely to account for a high proportion of use for the specific active ingredient. Based on the above exclusions, from a list of 47 medications that may contain phthalates, we *a priori* selected 4 for evaluation: *a*) mesalamine [DBP is contained in Asacol (Procter & Gamble Pharmaceuticals, Cincinnati, OH)]; *b* ) didanosine [DEP in Videx EC (Bristol Myers Squibb, Princeton, NJ)]; *c*) omeprazole [DEP in Prilosec (AstraZeneca, Wilmington, DE), and perhaps in generic versions there are unidentified high-molecular-weight phthalates such as diisooctyl, diisononyl, and diisodecyl phthalate]; and *d* ) theophylline [DEP in Theo-Dur and Uni-dur (Key Pharmaceuticals, Denilworth, NJ), and perhaps unidentified high-molecular-weight phthalates in generic versions].

We compared the mean urinary concentrations of each specific phthalate metabolite among users and nonusers of these medications. For medications with < 10 users, we used the Wilcoxon test for the crude comparison and also conducted a matched analysis by comparing each exposed subject with 5 randomly selected unexposed subjects matched on sex, age, race, and NHANES survey cycle. For more commonly used medications, we used linear regression models, both crude and adjusted for sex, age, race, and NHANES survey cycle. Use of sampling weights (NCHS 2007), transformation of phthalate metabolite concentrations using a natural logarithm, or adjustment for creatinine concentrations resulted in virtually the same results. All analyses were performed using SAS for Windows, version V.9.1 (SAS Institute Inc., Cary, NC).

## Results

Of the 31,112 individuals who responded to the demographic questionnaire, 7,999 had information on urinary phthalate concentrations. Overall, use of phthalate-containing medications increased with age and was similar for males and females; at least three exposed women were pregnant (Table 1).

Six subjects reported use of mesalamine; their individual MBP concentrations were 4,691, 4,358, 3,191, 1,055, 185, and 59 μg/L, and their corresponding creatinine-adjusted concentrations were 6,426, 4,150, 1,707, 1,160, 110, and 29.4 μg/g creatinine. The corresponding estimated doses of DBP for these six persons were 233, 151, 62, 42, 4, and 1 μg/kg/day. Thus, two of the six individuals exceeded the upper limit of the U.S. EPA RfD for DBP of 100 μg/kg/ day (U.S. EPA 2008), including a woman of childbearing age. The mean MBP concentration for mesalamine users was 2,257 μg/L, 50 times higher than the mean for nonusers (*p* < 0.0001); the mean concentrations of MCPP, a minor metabolite of DBP and also a metabolite of some other high-molecular-weight phthalates (Calafat et al. 2006), was about 10 times higher (Table 2). Compared with the 30 matched controls, for whom the mean MBP concentration was 24 μg/L, the mean MBP concentration for mesalamine users was almost 100 times higher (*p* < 0.001), and the mean concentration of MCPP was also higher (53 vs. 5 μg/L; *p*< 0.001).

**Figure 1:**
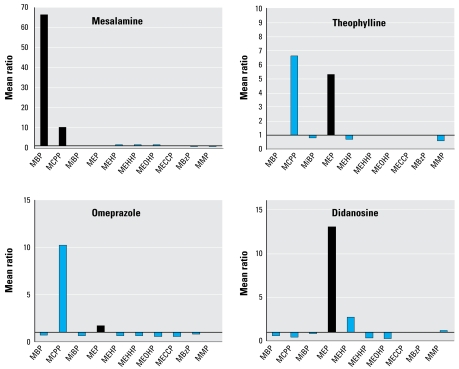
Mean ratio of creatinine-adjusted urinary concentrations of phthalate metabolites among users of specific medications compared with nonusers, NHANES 1999–2004. Abbreviations: MBzP, monobenzyl phthalate; MECCP, mono-(2-ethyl-5-carboxypentyl) phthalate; MEHHP, mono-(2-ethyl-5-hydroxyhexyl) phthalate; MEHP, mono-(2-ethylhexyl) phthalate; MEOHP, mono-(2-ethyl-5-oxohexyl) phthalate; MiBP, monoisobutyl phthalate; and MMP, monomethyl phthalate. Black bars indicate phthalates that might have been contained in each medication. The observed elevated MCPP concentrations for theophylline and omeprazole might result from the high-molecular-weight phthalates presumably contained as inactive ingredients in some generic versions.

As shown in Table 1 and Figure 1, users of didanosine, omeprazole, and theophylline had significantly higher urinary concentrations of MEP than did nonusers. For didanosine, the individual MEP concentrations for the three users were 11,950, 1,764, and 265 μg/L. The mean MEP concentration among didanosine users was 4,660 μg/L, substantially higher than the corresponding mean among the 15 matched controls (740 μg/L; *p* = 0.139). Mean concentrations of MCPP were also higher among users of omeprazole and theophylline compared with nonusers. For omeprazole and theophylline, crude and adjusted analyses gave similar results. Overall, the differences in mean concentrations for these two medications were due to a few exposed subjects with very high MEP concentrations rather than to an upward shift in the concentrations for all users. For omeprazole, 45% of users had MEP concentrations below the 50th percentile in the population, but 11% were above the 95th percentile (2,628 μg/L), with individual MEP concentrations of 4,219, 4,334, 4,821, 5,770, 6,242, 7,510, 8,374, 9,841, 11,159, and 18,292 μg/L. For theophylline, 37% of users had MEP concentrations below the 50th percentile in the population, but 22% were above the 95th percentile, with individual MEP concentrations of 5,101, 5,770, 10,096, 11,507, 12,821, and 23,810 μg/L.

The higher mean MEP concentration observed among mesalamine users was completely attributable to one patient taking theophylline and mesalamine simultaneously. Of note, urinary metabolite concentrations for phthalates not known to be included in the study medications did not differ between users and nonusers.

The interviewer observed the medicine container for all users of mesalamine and didanosine, and for most users of omeprazole (82.4%) and theophylline (81.5%); for the few subjects whose container was not seen at interview, phthalate metabolite urinary concentrations were elevated. All users of mesalamine, didanosine, and theophylline had been on the medication for at least 1 month. Two subjects had used omeprazole for < 1 week; their MEP concentrations were more than twice the average in the population.

## Discussion

Using data from NHANES 1999–2004, we confirmed the observation from an earlier case report (Hauser et al. 2004) that urinary concentrations of MBP, the major metabolite of DBP contained in Asacol, are significantly higher among users of mesalamine compared with nonusers. Four of six mesalamine users had estimated DBP intakes above or close to the upper limit of the doses suggested to be safe for the human population. Only one mesalamine user had urinary concentrations of MBP below the 95th percentile for the NHANES 1999–2004 population, which could possibly be explained by use of a medication containing mesalamine but not DBP (i.e., not Asacol). Alternatively, because the elimination half-life of DBP is measured in hours, if the patient missed any doses the days before the sample collection, the urinary concentrations of MBP would be expected to return rapidly to background levels (Koch et al. 2005). In addition, we found elevated urinary concentrations of MEP associated with use of didanosine, omeprazole, and theophylline—products that might contain the parent phthalate, DEP. The high MCPP concentrations found for some omeprazole and theophylline users might result from exposure to dioctyl phthalate or other unidentified high-molecular-weight phthalates (Calafat et al. 2006) potentially used in some formulations or packaging. The elevated levels found in two subjects who had been using omeprazole for less than 1 week suggest that just a few doses of this medication were enough to result in detectable elevations.

Of note, we found high urinary concentrations only for the metabolites of the phthalate diesters that might be present as inactive ingredients. Although all differences were statistically significant, the 50-fold difference for mesalamine was considerably larger than that for the other medications. Potential explanations include the following: *a*) patients with inflammatory bowel disease might have increased intestinal absorption of phthalates; *b*) Asacol is typically taken in doses of 6–12 tablets a day; *c*) the doses of phthalates per tablet might be higher in Asacol than in other medications; and *d*) most of the mesalamine use may have come from brands that contain DBP. The last explanation is consistent with the high MEP concentrations found for only some of the patients exposed to other medications, because presumably only some of the brands would have contained DEP.

Our study is limited by the inexact measures of medication use and subsequent likely misclassification of phthalate exposure. In addition to lacking information on dose and subject adherence, our data did not include information on brand names, so we could not restrict our assessment to active ingredients that also included a phthalate; rather, we only know that the active ingredient reported in the NHANES data could, in some formulations, contain a phthalate. Thus, we included as exposed those subjects who might have stopped their medications days before the urine sample collection or used a formulation or brand without phthalates or at least without the phthalates identified in this study. The finding of a few exposed subjects with very high phthalate concentrations rather than a generalized elevation for all users supports the likelihood that we incorrectly classified some nonexposed subjects as exposed. As a result, the associations we observed probably underestimate the true impact of exposure to phthalates in medications.

The present data suggest that certain medications can be an important source of exposure to some phthalates and that exposures from medications can far exceed population levels from various other sources. If our findings are confirmed, future risk assessments of environmental exposures to phthalates might need to consider high phthalate exposure in subjects on some of these medications. In some instances, medication use may contribute to human exposures that exceed the U.S. EPA RfDs (U.S. EPA 2008). Further exploration and consideration of the contribution of medications to phthalate exposure are warranted because of the potential for high delivered doses of phthalates to vulnerable segments of the population, particularly pregnant women or young children (Marsee et al. 2006).

## Figures and Tables

**Table 1:** Demographic characteristics of users and nonusers of specific phthalate-containing medications, NHANES 1999–2004.

		Medication [*n* (%)]			
Characteristic	Total no.	Mesalamine	Didanosine	Omeprazole	Theophylline
Total	7,996	6 (0.08)	3 (0.04)	91 (1.14)	27 (0.34)
Age (years)					
< 16	2,219	0 (0)	0 (0)	1 (0.05)	0 (0)
16–30	2,081	2 (0.10)	0 (0)	7 (0.34)	0 (0)
31–50	1,596	3 (0.19)	2 (0.13)	22 (1.38)	5 (0.31)
> 50	2,100	1 (0.05)	1 (0.05)	61 (2.90)	22 (1.05)
Sex					
Male	3,867	4 (0.10)	3 (0.08)	48 (1.24)	14 (0.36)
Female	4,129	2 (0.05)	0 (0)	43 (1.04)	13 (0.31)
Pregnant	319	1 (0.31)		2 (0.63)	0 (0)
Not pregnant	2,247	1 (0.04)		14 (0.62)	5 (0.22)
Pregnancy unknown	176	0 (0)		2 (1.14)	2 (1.14)
Missing	1,387	0 (0)		25 (1.80)	6 (0.43)
Race					
Mexican	2,154	1 (0.05)	2 (0.09)	11 (0.51)	4 (0.19)
Other Hispanic	352	0 (0)	0 (0)	4 (1.14)	1 (0.28)
Non-Hispanic white	3,212	4 (0.12)	0 (0)	58 (1.81)	17 (0.53)
Non-Hispanic black	1,983	0 (0)	1 (0.05)	13 (0.66)	5 (0.25)
Other	295	1 (0.34)	0 (0)	5 (1.69)	0 (0)
Education					
< High school	4,507	0 (0)	0 (0)	33 (0.73)	10 (0.22)
High school	1,271	2 (0.16)	0 (0)	22 (1.73)	8 (0.63)
> High school	2,206	4 (0.18)	3 (0.14)	35 (1.59)	9 (0.41)
Refused/unknown	12	0 (0)	0 (0)	1 (8.33)	0 (0)

**Table 2:** Number of exposed persons, mean urinary concentrations of phthalate metabolites (μg/L), and percentage of subjects above the 95th percentile (> 95th pct) in users of specific phthalate-containing medications and in nonusers, NHANES 1999–2004.

	Users of phthalate-containing medication^a^				
Metabolite	Nonusers	Mesalamine	Didanosine	Omeprazole	Theophylline
MBP					
No.	7,874	6	3	91	27
Mean	46.1	2,257**	16.8	28.2	199^b^
> 95th pct	5%	83%	0%	2%	7%
MCPP					
No.	5,365	6	2	54	16
Mean	5.3	52.6**	1.7	41.7**	44.6^b^**
> 95th pct	5%	67%	0%	35%	25%
MiBP					
No.	5,365	6	2	54	16
Mean	7.4	6.5	3.5	3.5	5.5
> 95th pct	5%	0%	0%	0%	6%
MEP					
No.	7,874	6	3	91	27
Mean	653	2,390^b^	4,660*	1,210*	2,924**
> 95th pct	5%	33%	33%	11%	22%
MEHP					
No.	7,874	6	3	91	27
Mean	9.3	12.2	14.5	5.6	4.7
> 95th pct	5%	0%	0%	3%	0%
MEHHP					
No.	5,365	6	2	54	16
Mean	57.9	82.3	10.8	32.3	50.5
> 95th pct	5%	17%	0%	4%	6%
MEOHP					
No.	5,365	6	2	54	16
Mean	37.3	50	5.6	20	34.2
> 95th pct	5%	17%	0%	4%	6%
MECCP					
No.	2,613	4	0	21	7
Mean	86.5	113.5	NA	35	45.5
> 95th pct	5%	25%		0%	0%
MBzP					
No.	7,874	6	3	91	27
Mean	37	16.5	22.4	24.8	22.3
> 95th pct	5%	0%	0%	4%	4%
MMP					
No.	5,365	6	2	54	16
Mean	5.3	2.9	4.2	5	2.1
> 95th pct	5%	0%	0%	9%	0%

Phthalates: MBP (*n* = 7,999); MiBP, mono-isobutyl phthalate (*n* = 5,439); MCPP (*n* = 5,439); MEP (*n* = 7,999); MEHP, mono-(2-ethylhexyl) phthalate (*n* = 7,999); MEHHP, mono-(2-ethyl-5-hydroxyhexyl) phthalate, (*n* = 5,439); MEOHP, mono-(2-ethyl-5-oxohexyl) phthalate, (*n* = 5,439); MECCP, mono-(2-ethyl-5-carboxypentyl) phthalate (*n* = 2,645); MBzP, mono-benzyl phthalate, (*n* = 7,999); MMP, mono-methyl phthalate, (*n* = 5,439). Sample sizes vary because not all phthalate metabolites were measured throughout the study period.

a *p*-Values from Wilcoxon test for mesalamine and didanosine and from linear regression models adjusted for creatinine, sex, age, NHANES cycle, mesalamine, and theophylline for omeprazole and theophylline.

b The differences in the mean MEP concentrations for mesalamine and MBP concentrations for theophylline are completely accounted for by one patient taking theophylline and mesalamine simultaneously. This patient was excluded from the MEP analysis for mesala-mine and from the MBP and MCPP analyses for theophylline.

* *p* = 0.05.

** *p* = 0.001.
